# Cortical auditory evoked potential in babies and children listeners^☆^

**DOI:** 10.1016/j.bjorl.2019.01.007

**Published:** 2019-02-22

**Authors:** Ana Carla Leite Romero, Ana Claudia Figueiredo Frizzo, Eduardo Federighi Baisi Chagas, Myriam de Lima Isaac

**Affiliations:** a Universidade Estadual Paulista (Unesp), Faculdade de Filosofia e Ciências (FFC), Campus de Marília, Marília, SP, Brazil; b Universidade de São Paulo (USP), Faculdade de Medicina de Ribeirão Preto, Ribeirão Preto, SP, Brazil

**Keywords:** Evoked potentials auditory, Electrophysiology, Auditory cortex, Infant, Child, Potenciais evocados auditivos, Eletrofisiologia, Córtex auditivo, Lactente, Criança

## Abstract

**Introduction:**

Cortical auditory evoked potentials have been increasingly used in research and audiological routines. However, there is a lack of studies with a large number of children who are stratified by age group. These would help clarify the variations in latency and amplitude of cortical auditory evoked potentials, and thus help establish reference values in children of different ages.

**Objective:**

To identify the variation in latency and amplitude of the cortical auditory evoked potentials and to establish reference values for the pediatric population.

**Methods:**

This was a cross-sectional study. Subjects were born at term and presented with no auditory complaints. A total of 105 children, of up to 6 years and eleven months old, who were divided into 7 age groups, named 1, 2, 3, 4, 5, 6 and 7, participated in the study. The tests were carried out using Biologic Navigator Pro. Initially, brainstem auditory evoked potential testing was performed in order to investigate the electrophysiological threshold of the subjects. Then, cortical auditory evoked potentials were elicited through oddball paradigm with tone burst differing in frequency, 750 Hz (frequent) and 1000 Hz (rare), and stimuli differing in speech: /ba/ (frequent) and /da/ (rare). In this study, descriptive and comparative analyzes of tonal and speech stimuli were performed for the age groups.

**Results:**

Significant differences were observed when comparing cortical auditory evoked potentials with speech stimulus in the right ear for P2 amplitude, for P1 latency the left ear, for P2 amplitude of the left ear; and for P1 amplitude of the left ear when performed with tonal stimuli.

**Conclusion:**

The obtained results can be considered as reference values of latency and amplitude of cortical auditory potentials in infants and children, and be used for monitoring their cortical auditory development.

## Resumo

**Introdução:**

Os potenciais evocados auditivos corticais têm sido cada vez mais usados em pesquisas e rotinas audiológicas. É importante que mais pesquisas sejam realizadas com maior número de crianças por faixa etária, a fim de contribuir para o conhecimento e descrever a variação das medidas de latência e amplitude, permitir estabelecer valores de referência desse potencial para crianças de diferentes grupos etários.

**Objetivo:**

Identificar a variação das medidas de latência e amplitude e estabelecer os valores de referência desse potencial para a população pediátrica.

**Método:**

Estudo do tipo transversal. Os indivíduos nasceram a termo, com ausência de queixas auditivas. Participaram do estudo 105 crianças de até 6 anos e 11 meses, divididas em 7 grupos de idade, denominados 1, 2, 3, 4, 5, 6 e 7. Os testes foram feitos com o Biologic Navigator Pro. Inicialmente, foi realizado o potencial evocado auditivo de tronco encefálico para investigar o limiar eletrofisiológico dos indivíduos. Em seguida, o potencial evocado auditivo cortical foi feito através do paradigma de *oddball* com *tone burst* diferindo em frequência, 750 Hz (frequente) e 1.000 Hz (raro) e o estímulo na fala: / ba/ (frequente) e /da/ (raro). Neste estudo, foram realizadas análises descritivas e comparativas dos estímulos tonais e de fala para os grupos etários.

**Resultados:**

Diferenças significantes foram observadas na comparação do potencial evocado auditivo cortical com estímulo de fala da orelha direita para amplitude de P2, para latência de P1 da orelha esquerda, para amplitude de P2 da orelha esquerda e para amplitude de P1 da orelha esquerda quando feita com estímulo tonal.

**Conclusão:**

Os resultados obtidos podem ser usados como medidas de referência de latência e amplitude dos potenciais auditivos corticais em lactentes e crianças, bem como para monitorar o desenvolvimento auditivo cortical.

## Introduction

Cortical auditory evoked potentials (CAEP) or late cortical evoked potentials have been increasingly used in research and audiological routines contributing to the diagnosis and monitoring of the auditory development in children.^1^, ^2^

These potentials reflect the neuroelectric activity of the primary and secondary auditory cortex and provide additional information about the biological processes involved in auditory processing; they can be used clinically to evaluate auditory maturation, auditory capacity and speech audibility in children with and without hearing loss.^3^, ^4^ There are few studies evaluating the functional significance of CAEP components in infants and it is yet not known whether the peaks observed at early ages are functionally similar to those found in adults.^5^

Several studies have used the P1 component of this potential to follow the cortical auditory development of children with cochlear implants and it has been observed that mostly there is a decrease in the latency of this component as the communicative behaviors (vocalizations), the speech and language skills, and the children's speech perception improve.^6^, ^7^

Studies have detected this potential in diverse populations of children, but there is still a lack of studies that show the values of CAEP during child development. Research with a large number of children, stratified by age group, can contribute to the diagnosis and monitoring of the maturation process of the auditory cortical structures. These studies may also bring new information that will assist in describing the variations in latency and amplitude of the CAEP, and in establishing reference values for children in different age groups.

Therefore this study aimed to identify these variations in latency and amplitude, and establish reference values in the pediatric population.

## Methods

This research project was submitted to the institutional research and ethics committee and approved (number 0701/2013).

This was a cross-sectional study. Subjects for this study were included based on the following inclusion criteria: subjects born at term, gestation and perinatal conditions without complications, adequate neuropsychomotor development, absence of auditory complaints at the presentation, electrophysiological threshold of 30 dBNA and latencies of waves I, III and V of the BAEP within the norms for the age group.

For this study, 105 subjects were randomly selected from 5 nurseries and 5 children's schools to ensure that the evaluated children could represent the general population. Meetings with the directors of participating nurseries and schools and legal guardians were held to explain the purpose and methodology of the study.

The selected subjects were aged between 0 and 6 years and 11 months and were distributed in the following groups:

Group 1: 15 subjects aged 0–11 months;

Group 2: 18 subjects aged 1 year to 1 year and 11 months;

Group 3: 13 subjects aged 2 years to 2 years and 11 months;

Group 4: 13 subjects aged 3 years to 3 years and 11 months;

Group 5: 16 subjects aged 4 years to 4 years and 11 months;

Group 6: 14 subjects aged 5 years to 5 years and 11 months;

Group 7: 16 subjects aged 6 years to 6 years and 11 months.

Brainstem auditory evoked potentials (BAEP) were recorded at the intensities of 80 dBNA and 30 dBHL in order to verify the integrity of the auditory pathways and to investigate the electrophysiological threshold.

BAEP and CAEP were recorded by the same examiner in all subjects using the equipment dual-channel auditory evoked potential Biologic Navigator®. Five disposable electrodes were used, the ground electrode was positioned at Fpz, the active electrodes at Fz and Cz in reference to the right (A2) and left (A1) lobe, using the two equipment recording channels. At the end of the collection, we chose to use the results from Cz, because they presented better morphology in the wave traces. The records at the threshold were repeated with the same number of measurements to ensure reproducibility of the examination.

The exams were performed in a room with acoustic treatment, with the subject lying down or sitting, according to the age group. The infants were in natural sleep and the children were in a state of quiet without the use of sedation.

Next, CAEP was carried out, in which the infants remained in natural sleep and the children in a quiet awake state watching a video (with no sound).

The record was made binaurally also through the use of five disposable electrodes. The ground electrode was positioned at Fpz, the active electrodes at Fz and Cz in reference to the right (A2) and left (A1) lobe, using the two recording channels of the equipment. At the end of the collection, we chose to use the results from Cz, because they presented better morphology in the wave traces.

The components were randomly searched in two scans, that is, first it was elicited by odball paradigm with the discrimination of tone burst that differed in tonal – (frequent stimulus: frequency of 750 Hz and rare stimulus: frequency of 1000 Hz)^8^ afterwards, for stimuli that differed for speech – (frequent stimulus: /ba/ and rare stimulus: /da/).

CAEP with speech stimuli were investigated using protocol.^9^ Natural speech stimuli were obtained in an acoustically treated room of the Laboratory of Linguistics. They were recorded on Praat® (Version 4.2.31), at 48 kHz, later recorded on CD for insertion in the format wave in the Biologic Navigator® Software. Initially, we worked on the contrast through the articulation point /ba/ – /da/. By the spectral definition and temporal arrangement, /ba/ was configured as the frequent stimulus and /da/ as the rare stimulus. The syllables [ba] and [da] were extracted from the emission of the words [ba’ba] and [da’da], respectively, corresponding to the second syllable. The duration of the syllables [ba] and [da] was 180 ms. The order and level of presentation of the stimulus were randomly processed by the software.

The two stimuli with different frequencies, and the speech stimuli were presented randomly at a rate of 1.1 stimuli per second, with a probability of 20% of rare stimulus from the total of 150 stimuli.

CAEPs were recorded by a trained evaluator with experience in electrophysiology. CAEP's wave marking was performed by two professionals with experience in electrophysiology and, when the tracing was considered difficult to analyze, that is, without agreement in relation to the marking, it was discussed among professionals, and the consensus was considered.

The identification of CAEP's waves was performed in the traces formed by the rare stimuli, following the criteria established in the literature.^10^

Quantitative variables were organized as mean ± standard deviation. The distribution of normality was verified by the Komorogov–Smirnov test. Levene test was used to test homogeneity of the variances. Anova-one-way test was applied to identify the differences between the means of the groups (*p* ≤ 0.05). Post Hoc LDS test was performed to indicate significant differences among groups, through similar overlapped letters (a, b, c, d).

For all analyzes, SPSS software version 19.0 for windows was used, adopting a significance level of 5%.

## Results

**Figure 1:**
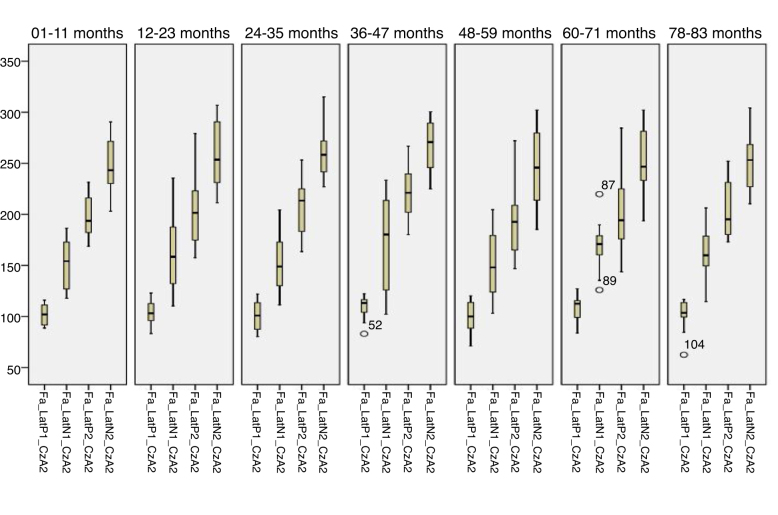
Minimum values, 1st quartile, 3rd quartile and maximum latency of Auditory Cortical Potentials according to each age group with speech stimulus in reference to CzA2.

**Figure 2:**
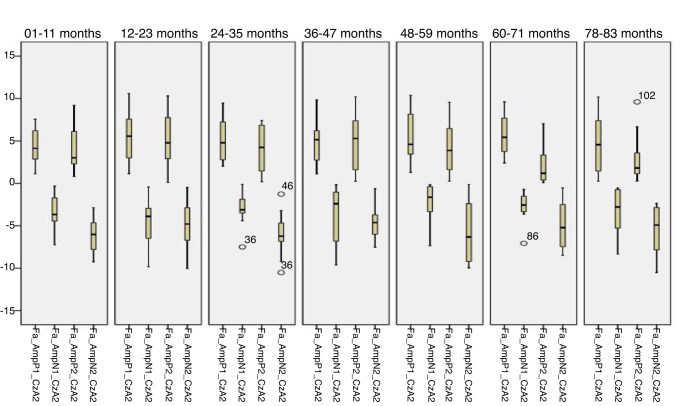
Minimum, 1st quartile, 3rd quartile and maximum amplitude values of Auditory Cortical Potentials according to each age group with speech stimulus in reference to CzA2.

**Figure 3:**
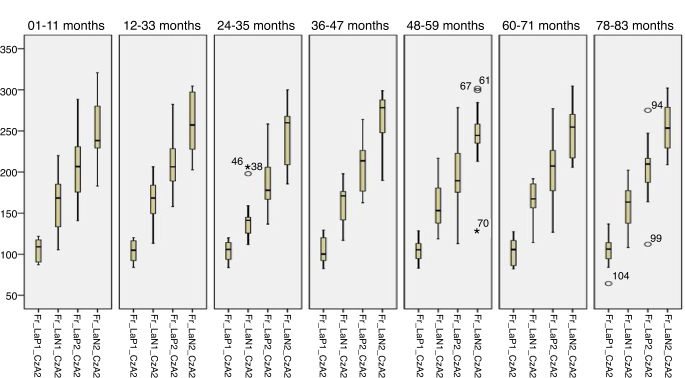
Minimum, 1st quartile, 3rd quartile and maximum latency values of Auditory Cortical Potentials according to each age group with tonal stimulus in reference to CzA2.

**Figure 4:**
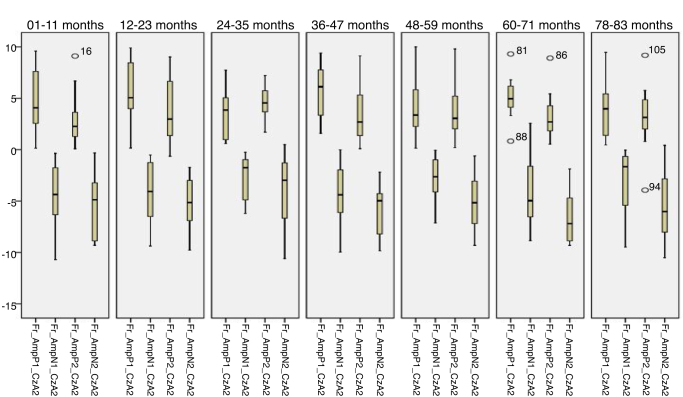
Minimum values, 1st quartile, 3rd quartile and maximum amplitude of Auditory Cortical Potentials according to each age group with tonal stimulus in reference to CzA2.

Figure 1, Figure 2 describes the minimum, 1st quartile, 3rd quartile and maximum latency values of CAEP according to each age group with speech stimulus in reference to CzA2; Figure 3, Figure 4 show the results with tonal stimuli.

**Figure 5:**
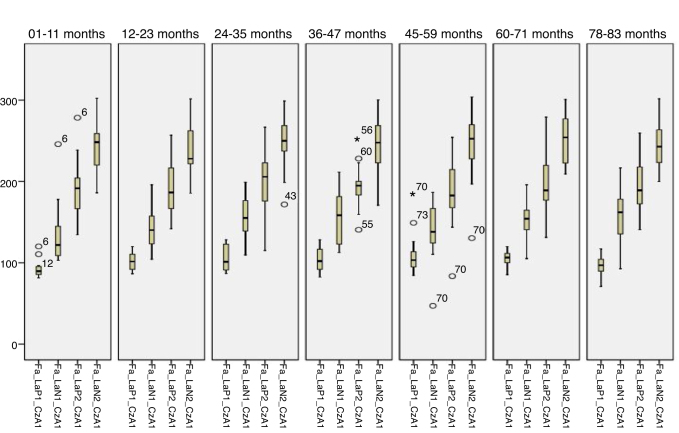
Minimum values, 1st quartile, 3rd quartile and maximum amplitude of Auditory Cortical Potentials according to each age group with speech stimulus in reference to CzA1.

**Figure 6:**
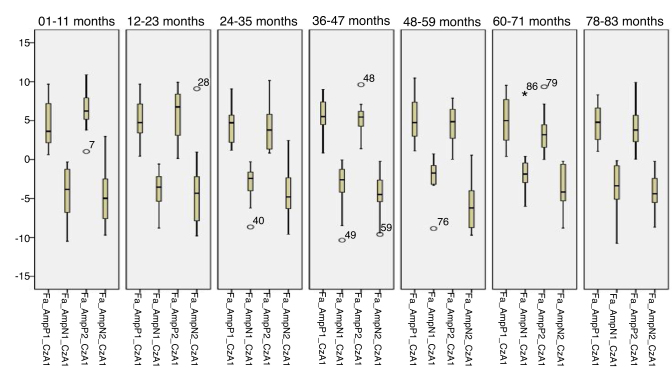
Minimum values, 1st quartile, 3rd quartile and maximum amplitude of Auditory Cortical Potentials according to each age group with speech stimulus in reference to CzA1.

**Figure 7:**
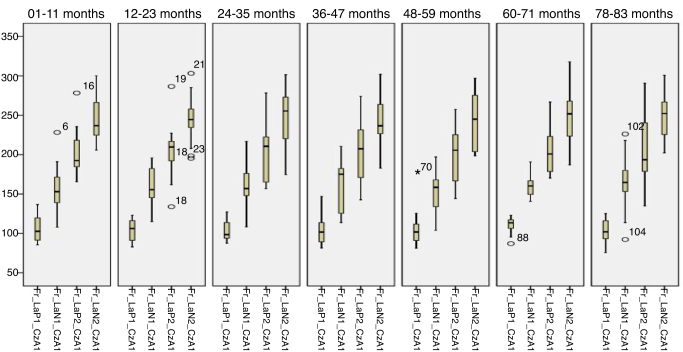
Minimum values, 1st quartile, 3rd quartile and maximum amplitude of Auditory Cortical Potentials according to each age group with tonal stimulus in reference to CzA1.

**Figure 8:**
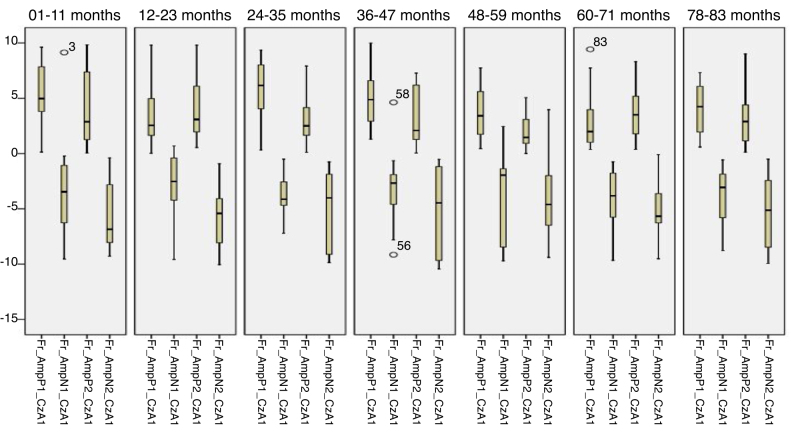
Minimum values, 1st quartile, 3rd quartile and maximum amplitude of Auditory Cortical Potentials according to each age group with tonal stimulus in reference to CzA1.

Figure 5, Figure 6 describes the minimum, 1st quartile, 3rd quartile and maximum latency values of CAEP for each age group with speech stimuli in reference to CzA1, Figure 7, Figure 8 with tonal stimuli.

Table 1 shows the mean values, standard deviation and *p*-value of CAEP with speech stimulus in reference to CzA2 for each age group. Statistically significant differences were observed with a decrease in wave amplitude as age increased only for the P2 amplitude of Groups 1, 2, 4, 6 and 7.

**Table 1:** Mean, standard deviation and *p* value of Auditory Cortical Evoked Potential for latency and amplitude of P1, N1, P2 and N2 with each age group in reference to speech stimulus CzA2.

	Groups							*p*
	1	2	3	4	5	6	7	
LAT P1	101.8 ± 10.0	103.7 ± 11.9	101.9 ± 14.4	109.0 ± 11.5	100.3 ± 14.7	108.0 ± 13.2	102.7 ± 13.9	0.47
AMP P1	4.4 ± 2.0	5.6 ± 2.8	5.0 ± 2.3	4.9 ± 3.0	5.5 ± 2.7	5.6 ± 2.3	4.6 ± 3.3	0.80
LAT N1	151.0 ± 24.3	159.4 ± 34.1	152.8 ± 31.1	170.6 ± 44.1	150.3 ± 33.5	168.2 ± 24.1	162.7 ± 23.8	0.40
AMP N1	3.3 ± 1.7	4.3 ± 2.6	2.9 ± 1.8	3.7 ± 3.4	2.2 ± 2.2	2.6 ± 1.5	3.1 ± 2.4	0.20
LAT P2	197.5 ± 19.1	203.7 ± 36.4	206.9 ± 27.6	221.6 ± 26.5	194.1 ± 37.5	202.4 ± 36.5	203.7 ± 28.1	0.36
AMP P2	4.1 ± 2.7^a^	5.3 ± 3.0^b^	4.0 ± 2.8	5.0 ± 3.6^c,d^	4.1 ± 2.9	2.0 ± 2.2^a,b,c^	2.7 ± 2.4^b,d^	0.02^a^
LAT N2	247.9 ± 26.5	259.1 ± 32.7	262.1 ± 26.1	266.7 ± 27.1	244.8 ± 38.2	255.6 ± 33.9	251. 6 ± 29.4	0.47
AMP N2	6.2 ± 2.1	4.6 ± 2.5	5.9 ± 2.4	4.5 ± 2.0	5.6 ± 3.6	4.7 ± 2.6	5.6 ± 2.9	0.48

a *p* ≤ 0.05 statistical difference between groups (Anova-one-way). Equivalent overlapping letters (a, b, c, d) indicate significant difference between the groups by the Post Hoc LSD test.

Table 2 describes these findings in reference to CzA1 for each age group. Statistically significant differences were observed for the P1 latency of Groups 1, 3, 4, 5, 6 and 7 with lower latency time for subjects from 0 to 12 months of age when compared with the subjects of 3, 4, 5 and 6 years, followed by a further decrease in latency for subjects at 7 years and for the P2 amplitude of Groups 1, 2, 3, 5, 6 and 7 with a decrease of wave amplitude as the subjects’ age increased.

**Table 2:** Mean, standard deviation and *p* value of Auditory Cortical Evoked Potential for latency and amplitude of P1, N1, P2 and N2 with each age group in reference to speech stimulus CzA1.

	Groups							*p*
	1	2	3	4	5	6	7	
LATP1	91.9 ± 10.4^a,b,c,d^	101.5 ± 10.5	106.7 ± 16.5^a^	103.1 ± 15.6^b^	110.0 ± 25.5^c,e^	105.1 ± 9.7^d^	96.5 ± 11.7^e^	0.02^a^
AMPP1	4.3 ± 2.8	5.3 ± 2.6	4.1 ± 2.5	5.9 ± 2.3	5.2 ± 2.7	5.1 ± 2.88	4.7 ± 2.4	0.72
LATN1	134.7 ± 37.9	142.6 ± 22.7	155.3 ± 27.3	152.9 ± 32.8	139.4 ± 33.9	153.0 ± 23.8	157.3 ± 32.7	0.27
AMPN1	4.2 ± 3.2	3.9 ± 2.4	3.0 ± 2.3	3.4 ± 3.1	2.1 ± 2.1	1.3 ± 3.3	3.5 ± 3.1	0.08
LATP2	189.1 ± 36.4	190.5 ± 32.1	201.4 ± 41.6	194.8 ± 29.2	186.6 ± 42.3	198.8 ± 40.3	193.9 ± 34.7	0.93
AMPP2	6.3 ± 2.4^a,b,c,d^	5.6 ± 3.2^e^	3.9 ± 2.7^a^	5.4 ± 1.9	4.3 ± 2.5^b^	3.4 ± 2.4^c,e^	3.9 ± 2.6^d^	0.02^a^
LATN2	245.0 ± 32.8	238.9 ± 34.5	249.9 ± 37.0	246.7 ± 35.7	245.0 ± 41.2	252.9 ± 32.1	244.0 ± 29.0	0.95
AMPN2	4.9 ± 3.7	4.1 ± 4.5	4.6 ± 3.5	4.7 ± 2.9	5.7 ± 3.1	3.7 ± 2.6	4.3 ± 2.3	0.76

a *p* ≤ 0.05 *statistical* difference between groups (Anova-one-way). Equivalent overlapping letters (a, b, c, d) indicate significant difference between the groups by the Post Hoc LSD test.

Table 3 shows the mean, standard deviation and *p* value of CAEP with tonal stimuli in reference to CzA2 for each age group. No statistically significant differences were observed.

**Table 3:** Mean, standard deviation and *p* value of Auditory Cortical Evoked Potential for the latency and amplitude of P1, N1, P2 and N2 with each age group with respect to CzA2 with tonal stimulus.

	Groups							*p*^a^
	1	2	3	4	5	6	7	
LATP1	106.0 ± 13.0	104.0 ± 12.0	104.0 ± 12.1	105.5 ± 16.6	104.1 ± 12.9	103.4 ± 15.9	103.6 ± 16.6	0.99
AMPP1	4.8 ± 3.1	5.6 ± 2.9	3.4 ± 2.3	5.4 ± 2.6	4.1 ± 2.7	5.0 ± 1.9	3.9 ± 2.8	0.23
LATN1	163.7 ± 32.5	168.0 ± 24.4	144.3 ± 28.4	164.1 ± 23.1	157.5 ± 27.1	165.2 ± 23.7	160.9 ± 27.4	0.31
AMPN1	4.5 ± 3.20	3.9 ± 2.8	2.6 ± 2.1	4.1 ± 3.01	2.9 ± 2.1	4.1 ± 3.4	2.9 ± 3.1	0.42
LATP2	209.1 ± 40.9	210.3 ± 35.5	192.0 ± 37.3	212.5 ± 33.6	195.8 ± 39.5	200.7 ± 41.8	202.6 ± 36.2	0.73
AMPP2	2.9 ± 2.4	3.7 ± 3.0	4.4 ± 1.7	3.5 ± 2.7	3.6 ± 2.4	3.2 ± 2.1	3.2 ± 2.8	0.76
LATN2	249.9 ± 37.3	256.7 ± 25.9	243.5 ± 25.9	265.2 ± 30.5	244.9 ± 39.3	252.3 ± 34.1	254.4 ± 31.0	0.69
AMPN2	5.4 ± 3.1	5.0 ± 2.3	4.3 ± 3.7	5.8 ± 2.7	4.9 ± 2.6	6.6 ± 2.4	5.4 ± 3.3	0.52

a *p* ≤ 0.05 statistical difference between groups (Anova-one-way).

Table 4 describes these findings in reference to CzA1 for each age group. A statistically significant difference was observed in the P1 amplitude of Groups 1, 2, 3, 5 and 6 in which the subjects presented a decrease of amplitude from the 5 years.

**Table 4:** Mean, standard deviation and *p* value of the Auditory Cortical Evoked Potential for the latency and amplitude of P1, N1, P2 and N2 with each age group with respect to CzA1 with tonal stimulus.

	Groups							*p*
	1	2	3	4	5	6	7	
LATP1	105.5 ± 15.8	103.8 ± 14.0	103.1 ± 12.8	104.3 ± 17.8	105.6 ± 23.1	110.6 ± 10.0	103.7 ± 13.9	0.89
AMPP1	5.4 ± 2.9^a,b^	3.5 ± 2.8^a,c^	5.8 ± 2.8^c,d,e^	5.0 ± 2.7	3.5 ± 2.2^d^	3.0 ± 2.7^b,e^	4.0 ± 3.3	0.03^a^
LATN1	156.6 ± 29.5	160.1 ± 26.1	159.3 ± 28.5	162.9 ± 32.4	154.0 ± 28.8	161.4 ± 14.2	165.0 ± 34.2	0.95
MPN1	3.3 ± 4.4	3.1 ± 3.0	3.8 ± 1.8	3.1 ± 3.4	3.8 ± 3.8	4.3 ± 3.1	4.0 ± 2.7	0.93
LATP2	202.2 ± 29.9	204.3 ± 31.9	203.8 ± 38.5	207.2 ± 42.5	201.0 ± 36.4	203.5 ± 27.6	206.1 ± 43.7	0.99
AMPP2	4.0 ± 3.5	4.1 ± 3.1	3.3 ± 2.7	3.3 ± 2.6	2.0 ± 1.5	3.6 ± 2.5	3.1 ± 2.4	0.38
LATN2	244.6 ± 29.4	243.4 ± 27.5	250.4 ± 40.6	240.5 ± 38.8	243.9 ± 38.2	248.5 ± 32.8	249.1 ± 28.9	0.98
AMPN2	5.6 ± 2.9	5.9 ± 2.5	5.0 ± 3.6	5.5 ± 4.0	4.2 ± 3.3	5.0 ± 2.6	5.3 ± 3.1	0.81

a *p* ≤ 0.05 statistical difference between groups (Anova-one-way). Equivalent overlapping letters (a, b, c, d, e) indicate significant difference between the groups by the Post Hoc LSD test.

## Discussion

Initially, the findings of this study were described in Figure 1, Figure 2, Figure 3, Figure 4, Figure 5, Figure 6, Figure 7, Figure 8 from the values of minimum, maximum, 1st Quartile (25%) and 3rd Quartile (75%) of CAEP's latency and amplitude, with tonal speech stimuli.

If we perform a more qualitative analysis of the results, we can observe that, although discrete, there is a variation of latency mainly in relation to the P1 component, in which a decreased latency was observed for Group 1, followed by a stable period, and a new decrease for the Group 7 that presented even shorter latencies in comparison to the others. These findings may be explained by neural budding, in which cell growth occurs from axons and is characterized by a rapid initial phase followed by a slower phase which last for months, as observed in this study.^11^

Some studies have found that P1 latencies remained stable between 0 and 6 years and reported that this finding is consistent with the maturation of neural generators of P1 in the primary auditory cortex,^12^ while some reported that only after 5 years was a decrease in the latency of P1 observed.^1^, ^2^, ^5^

Authors^5^ suggest that P1 reflects the propagation of activity across the deeper layers of the auditory cortex, and at the end of childhood, between 6 and 12 years, the final stage of structural maturation of the auditory cortex occurs when axons mature and become equivalent to those of an adult, so the latencies of the P1 component would only begin to show greater decrease at around 6 years of age, as observed in our study.

A comparison of CAEP with each age group was then performed using mean values, standard deviation and *p* value in reference to CzA2 and CzA1 with speech and frequency stimulus of each group.

Table 1 showed the CAEP findings with speech stimuli in reference to CzA2, and a significant difference was observed among the age groups for the P2 amplitude, with a decrease of the wave amplitude as there was an increase in age; such findings demonstrate the occurence of a maturation process of the structures involved in the generation of this component.^13^ In addition, studies^14^ state that the P2 wave has generators in several regions of the primary, secondary and reticular auditory cortex, which are associated with the attention the individual gives to the sound stimulus and with the inhibition of the processing of competitive stimuli, as maturation of the auditory pathways occurs; the subjects need less auditory attention to discriminate the stimuli and adapt to them or ignore them, generating a wave of smaller amplitude.^13^, ^15^

Table 2 reveals a significant difference among the age groups for the P1 latency of CzA1. These results demonstrate a shorter latency for subjects from 0 to 12 months of age when compared with subjects of 3, 4, 5 and 6 years, followed by a further decrease in latency for subjects at 7 years of age. Authors^16^ conducted a study examining the maturation of CAEPs from 3 months of age to 8 years of age and observed that only the P1 peak was clearly present, it was the most robust peak and was easily identifiable in all ages.

One study^17^ observed that the P1 latency did not vary significantly in childhood or early childhood, that is, during the first 6 years of life, remaining around 92.1 ms, which corroborates the findings of this study.

Authors performing cortical potentials in cochlear implanted children^6^, ^7^ reported that the hearing impaired children have an increase in P1 latency pre cochlear implant, and that after use of the implant, there is a very fast activation of the P1 component, with lower latency values correlating with a longer duration of device usage. In addition, those authors also observed that the development of the P1 component of children after cochlear implantation follows the same pattern as a normal hearing child, but with a delay in the maturational process^18^ It was also observed that the P2 amplitude of Groups 1, 2, 3, 5, 6 and 7 presented a decrease in wave peaks as the subjects’ age increased. Researchers^19^, ^20^ have concluded that peak wave amplitude reflected synaptic density in the primary auditory cortex, and in the first 3 months of life it may appear with double peaks. As age increases, auditory maturation takes place and neural synchrony improves, which result in a higher mean response with a single peak that should decrease amplitude.^1^, ^5^

In Table 4, a significant difference was observed among the age groups for the P1 amplitude of CzA1 with tonal stimulus. It was verified that the subjects presented decreased amplitude after age 5 years. As mentioned, CAEP's amplitude tend to decrease as auditory maturation occurs. In addition, authors^17^ observed that the P1 peak amplitude decreased systematically after the age of 1 year, and were twice as large as that of adults. The literature^21^ also indicated that younger children tend to show relatively higher peak amplitudes that should decrease with adulthood.

One study^22^ discussed the difficulty in recording CAEPs and postulated that it was due to the absence of a normality pattern and the lack of routine format for this examination in the clinical practice among the professionals, in addition to several factors ranging from electrodes placement to the physiological issues of the individual, which may interfere with the acquiring these potentials.

Authors^10^ investigated the stability in the analysis and interpretation of CAEP following a set of predetermined criteria and pointed out the need for appropriate examiner training, as well as the use of well-established criteria for the analysis of the tracing so that the interpretations are more accurate and the results reliable.

It is important to highlight that, in this study, in order to guarantee the reliability of the results, the data were collected and analyzed by a trained and experienced examiner who followed the criteria proposed by previous studies.^10^ In addition, for this test to be useful in clinical practice, it is necessary for the examiners to have a protocol with pre-established normative latency and amplitude values for all age groups, which justifies the scientific contribution of this study.

Therefore, these results can be used as reference measures of CAEP latency and amplitude of infants and children, and used to monitor the cortical auditory development of those with audiological alterations submitted to speech therapy with the use of hearing aids.

## Conclusion

When comparing the age groups, significant differences were observed for speech stimuli in reference to CzA2 for P2 amplitude, for speech P1 latency in CzA1, for speech P2 amplitude in CzA1 and for P1 amplitude of CzA1 when performed with tonal stimulus.

Furthermore, in this study, we verified that is possible to record CAEP in infants and children aged 0–6 years and 11 months, since it was present in all the analyzed subjects.

Therefore, these results can be used as reference measures of CAEP latency and amplitude in infants and children, and for the monitoring of cortical auditory development of those with audiological alterations submitted to speech therapy with the use of hearing aids.

## Conflicts of interest

The authors declare no conflicts of interest.
